# Supplementary material for: A generative force model for surgical skill quantification using sensorised instruments
Source: Commun Eng. 2023 Jun 10;2:36. doi: 10.1038/s44172-023-00086-z (PMC10955904; doi:10.1038/s44172-023-00086-z)
Supplement: Supplementary file 2 — Supplementary Information PDF [file 44172_2023_86_MOESM2_ESM.pdf]

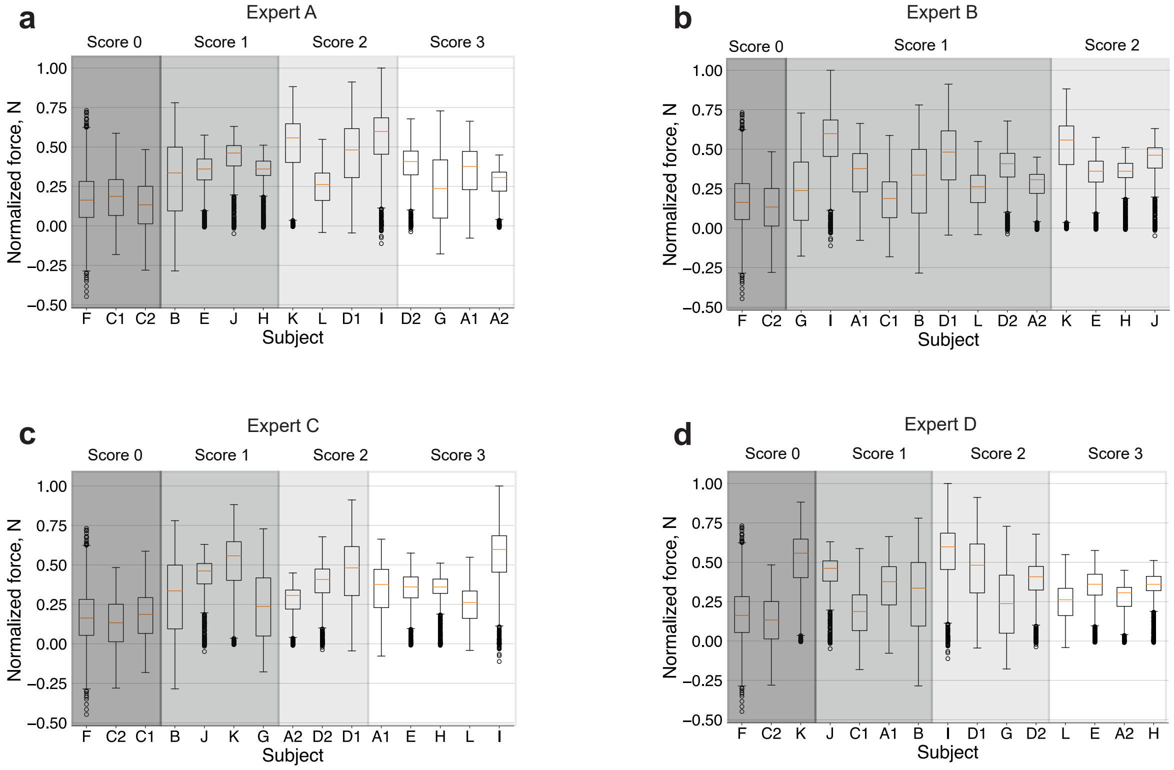

Supplementary Figure 1: **Box plots of force samples (N = 1,440) sorted by expert evaluation scores.** Subjective evaluation by surgical experts (Supplementary Table 1). Proficiency is scored from lowest (0, darkest region) to highest (3, lightest region).

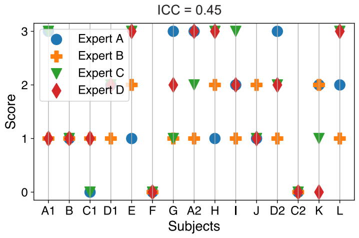

Supplementary Figure 2: **Expert score across the subject trials**. The intraclass correlation coefficient (ICC) is 0.45 (N = 15 independent trials).

Supplementary Table 1: **Expert evaluation (scoring from 0 to 3) of task execution based on video of the trials.** Note: Matching scores among experts are highlighted in bold. The original scoring by expert B (shown in parentheses) was normalised to a 0-3 scale from provided 0-5 scale, see Supplementary Table 3 for expert's comment.

Subject	Expert A	Expert B	Expert C	Expert D
A (1st trial)	3	1	3	1
A (2nd trial)	3	1 (2)	2	3
B	**1**	**1** (2)	**1**	**1**
C (1st trial)	0	1	0	1
C (2st trial)	**0**	**0**	**0**	**0**
D (1st trial)	2	1 (2)	2	2
D (2nd trial)	3	1 (2)	2	2
E	1	2 (3)	3	3
F	**0**	**0**	**0**	**0**
G	3	1	1	2
H	1	2 (3)	3	3
I	1	1	3	2
J	1	2 (3)	1	1
K	2	2 (3)	1	0
L	2	1 (2)	3	3

Supplementary Table 2: **Expert A supplementary comment.**

Subject	Comments
A (1st trial)	Good even pressure, skin traction, continuous movement.
A (2nd trial)	Good movement, gentle and continuous.
B	Nice flow.
C (1st trial)	Minimal, if any meaningful traction. Movement with knife is not fluid. Seems to be dragging/digging with knife rather than cutting.
C (2st trial)	Awkward saw-like movement of blade.
D(1st trial)	Nice fluid movements.
D (2nd trial)	-
E	Good movement of blade, but awkward movement at wrist.
F	Digging at the material, appears to be sawing through it. Traction is not meaningful.
G	Nice grip and knife movement, fluid re-positioning of fingers for traction.
H	Fluid movement but limited use of hands for traction.
I	Good, but odd grip.
J	Slightly dragging the knife through the material, little traction.
K	Fluid, but limited traction and very firm grip of blade without optimising the use of wrist/hand movements.
L	-

Supplementary Table 3: **Expert B supplementary comment.**

Score	Comments
0	Unable to perform dissection or rough/uneven scalpel use.
1	Able to cut through skin with some care.
2	As 1 + smooth scalpel strokes (did not go over the same area twice, for the same level, nor jerky movement) + uses supporting hand to provide tension.
3	As 2 + held blade 90 degrees to incision line (no bevelling).
4	As 3 + recognises that the epidermal layer should be scored (smooth light cut) first, allowing a smoother dissection of deeper layers.
5	Perfect dissection (all of the above).

Supplementary Table 4: **Expert D supplementary comment.**

Subject	Comments
A (1st trial)	Good use of non-dominant hand, accurate in centre of dots for first two but that accuracy decreased from 3rd ellipse onwards, slightly bevelled cut because knife not at right angles to skin.
A (2nd trial)	Smooth single glide on each half of ellipse, accurate, excellent use of non-dominant hand, good knife angle, confident, very slight bevelling on the first part of lower incision of the ellipse.
B	Fairly consistent accuracy in centre of dots, knife angle OK, less use of non-dominant hand, there seemed to be less need for use of non-dominant hand, bevelling present.
C (1st trial)	More of a stabbing/sawing, sometimes jerky movement, rather than knife gliding over skin. Looks like the operator cut more deeply into the model and there was more friction on the knife, became smoother and quicker from 1-6, good accuracy
C (2st trial)	Stabbing, sawing movements, deep cuts, with possible variation in depth of cut, no glide, accuracy medium to poor, no bevel, ragged edges, knife slipped once, non-dominant hand used well.
D (1st trial)	Fairly deep cuts, smoother action (knife seems sharper), longer continuous knife sweeps, seemed more confident and quicker, accuracy good, except at corners, no bevelling. Top layer of ”skin” seemed much more mobile in this specimen. (In marked contrast to subject H) I think this made the incisions more difficult, especially in the first 2 ellipses - leading to ragged edges, where the top layer seemed to be dragged by the knife rather than a clean cut.
D (2nd trial)	Confident glide, non-dominant hand quality good, cuts deeper at start of incision, seemed more superficial at end of each incision, accuracy medium.
E	More shoulder/trunk movements than others, hand obscured view more than in other recordings, no bevelling, knife held more vertically in line of incision possibly resulting in a deeper cut and less glide, but knife glided fairly well with no re-cuts.
F	Non-pencil grip, deep cuts, sawing action, little/no use of non-dominant hand to stabilise skin, accuracy poor, no bevelling.
G	Shorter glides, accuracy OK, no bevelling, good knife angle, more superficial incisions.
H	Confidence, smooth glide single glide for each side of ellipse, limited (need to) use non-dominant hand, good knife angle, accuracy fine, on last side of last ellipse near end - lateral pressure to correct course led to slight ragged edge. Top layer of skin seemed very firmly attached to lower layers with no sheering.
I	OK, but less clean glide of knife, some breaks in continuity of glide, pushing knife to the side to correct inaccurate course, very occasional bevel and slight dragging of top layer of skin which was not as well attached to deeper layer, accuracy medium to poor.
J	Less good use of non-dominant hand (interestingly, non-dominant hand often applied tension along the long axis of the ellipse rather than at right angles to the incision) knife did not glide smoothly, leading to dragging of top layer of skin, with some ragged cuts, no significant bevelling, knife more vertical and made deeper cuts, which may have contributed to the jerky movements while cutting against greater resistance.
K	Accuracy is very poor (missed dots and overshot corners), non-dominant hand use could have been better - did not seem to apply much stretch, side pressure to correct inaccurate course, leading to ragged edges, e.g. ellipse no 2.
L	Non pencil grip, confident although not a single smooth glide, good use of non-dominant hand, some lateral force to correct course, but this was minimal, accuracy - medium.

Supplementary Table 5: **Expert grading of phantom realism (from least to most realistic. H - Hard, S - Soft and VS - Very soft)**

Realism	Bottom layer	Middle layer	Top layer	Comments
1	H (20 ml)	N/A	H (25 ml)	Not very realistic
2	S (20 ml)	N/A	S (25 ml)	N/A
3	S (20 ml)	N/A	H (25 ml)	N/A
4	S (20 ml)	VS (20 ml)	H (20 ml)	Child-like skin
5	H (25 ml)	VS (20 ml)	H (20 ml)	An older adult's skin
6	S (25 ml)	VS (50 ml)	S (20 ml)	Realistic
7	S (25 ml)	VS (20 ml)	S (20 ml)	Very realistic

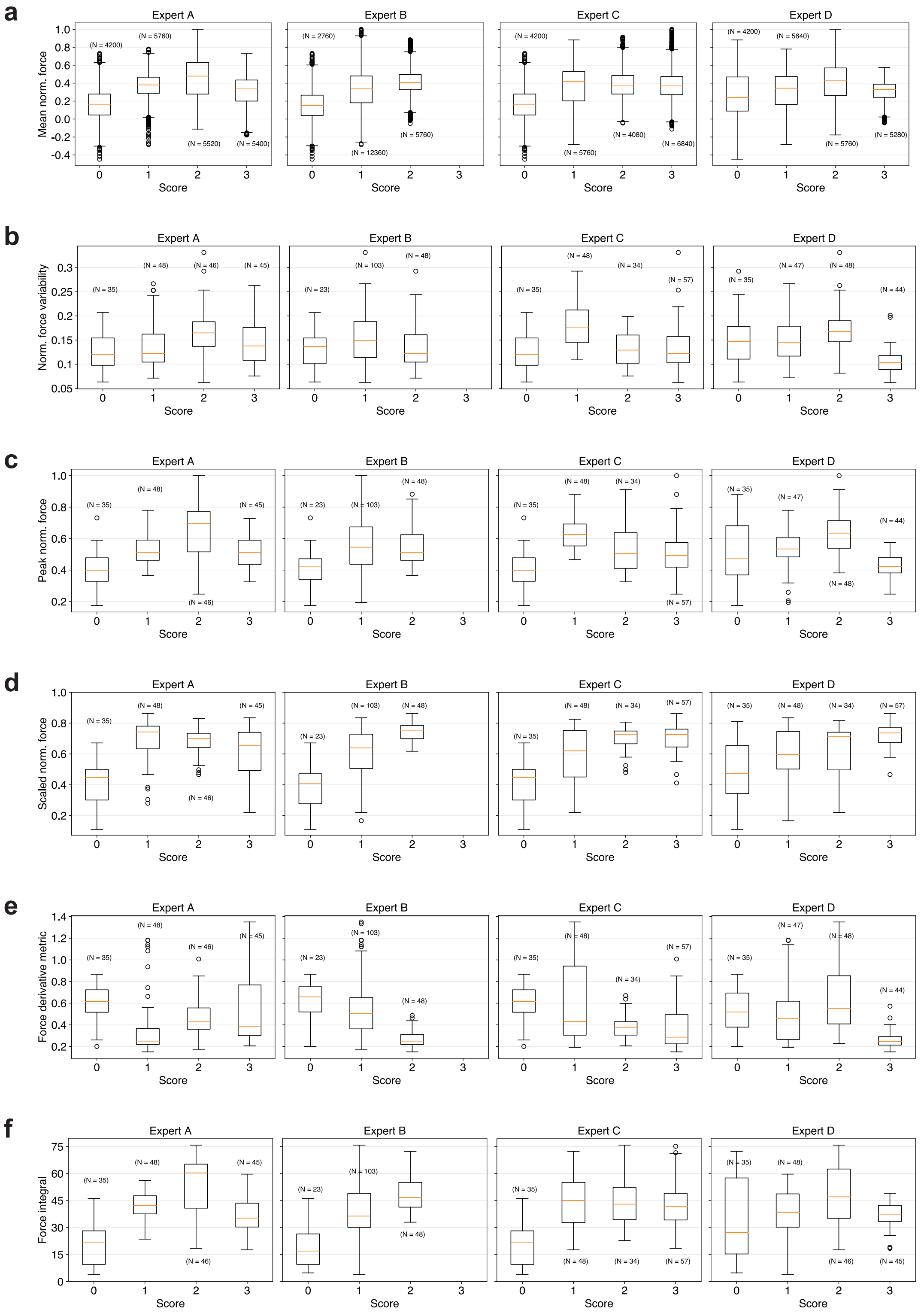

Supplementary Figure 3: **Box plots of force-based metrics across different expert ratings**. **a** Mean normalised force. **b** Normalised force variability. **c** Peak normalised force. **d** Scaled normalised force. **e** Force derivative metric. **f** Force integral metric.

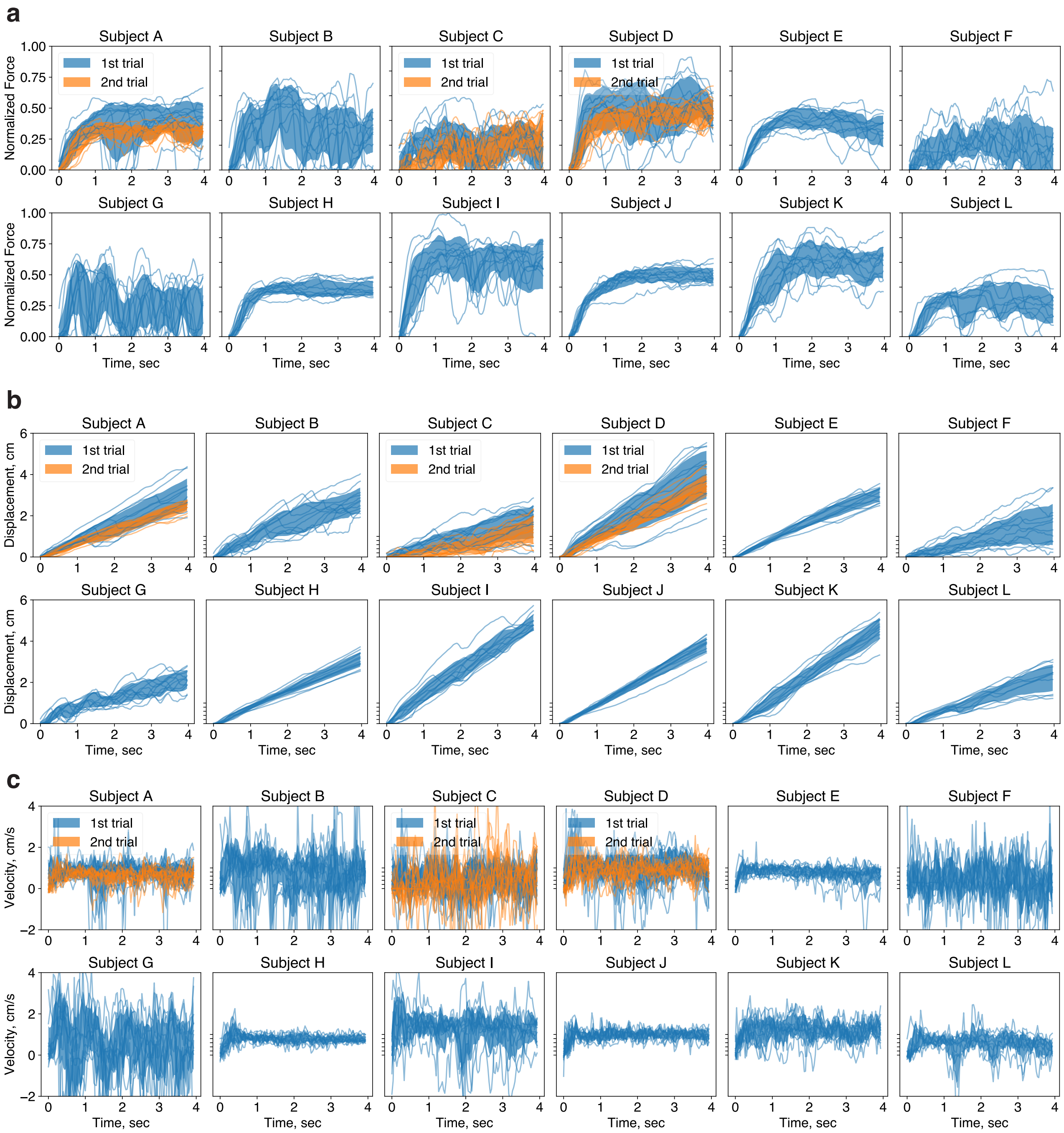

Supplementary Figure 4: **Data used for training the elliptical excision force model.** **a** Normalised measurements of excision forces. **b** Blade's virtual displacement traces. **c** Blade's virtual velocity traces. Note: the individual traces are shown as semi-transparent lines and their distribution (one standard deviation) is shown as coloured areas. For each distribution, there is $N = 12$ independent trials.

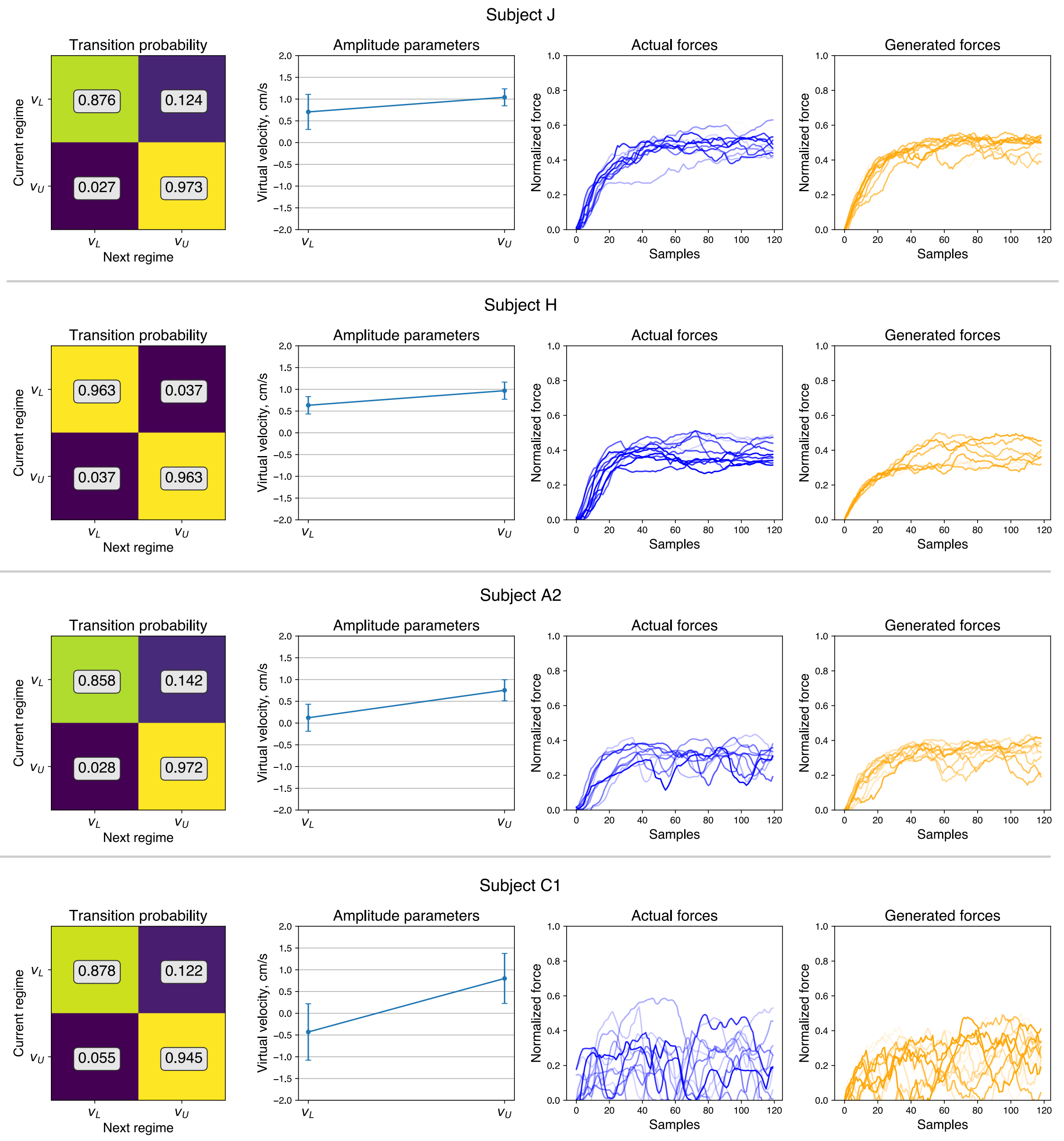

Supplementary Figure 5: **The effect of model parameters on temporal and amplitude characteristics of learned excision behaviour.** Note: In the Transition probability matrix, the brighter colour represents the higher probability. In the Amplitude parameters plot, the error bars represent the standard deviation of virtual velocity values.

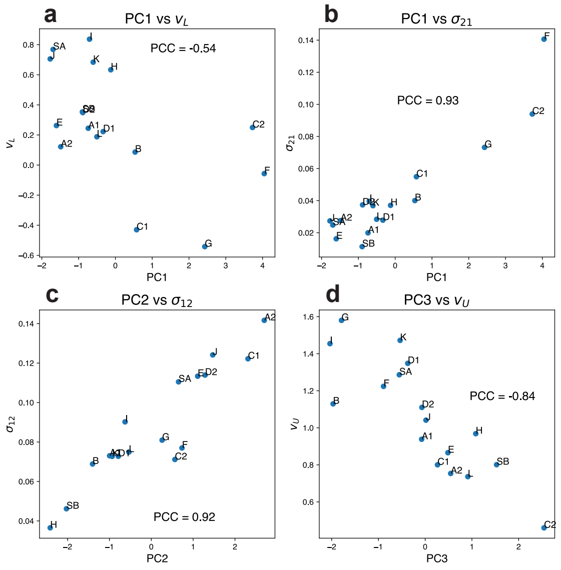

Supplementary Figure 6: **Correlation between principal components PC1, PC2 and PC3 and model parameters v_L, v_U, σ_{12} and σ_{21} (N = 17 independent trials).** Note: PCC - Pearson Correlation Coefficient. Letters A to L correspond to medical students (where numeral indicates the trial), "SA" and "SB" correspond to surgeon A and B, respectively.

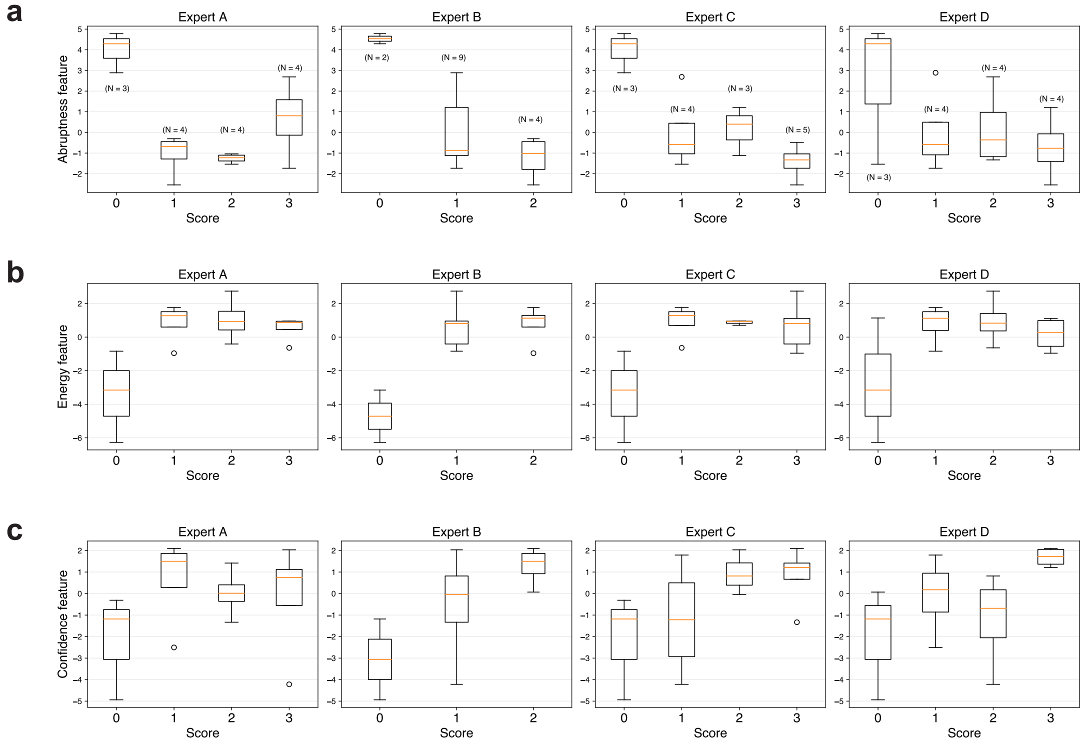

Supplementary Figure 7: **Learned features and expert rating. a** Abruptness feature, **b** Energy feature and **c** Confidence feature. Note: the sample size for each evaluation group is shown in pane **a**.

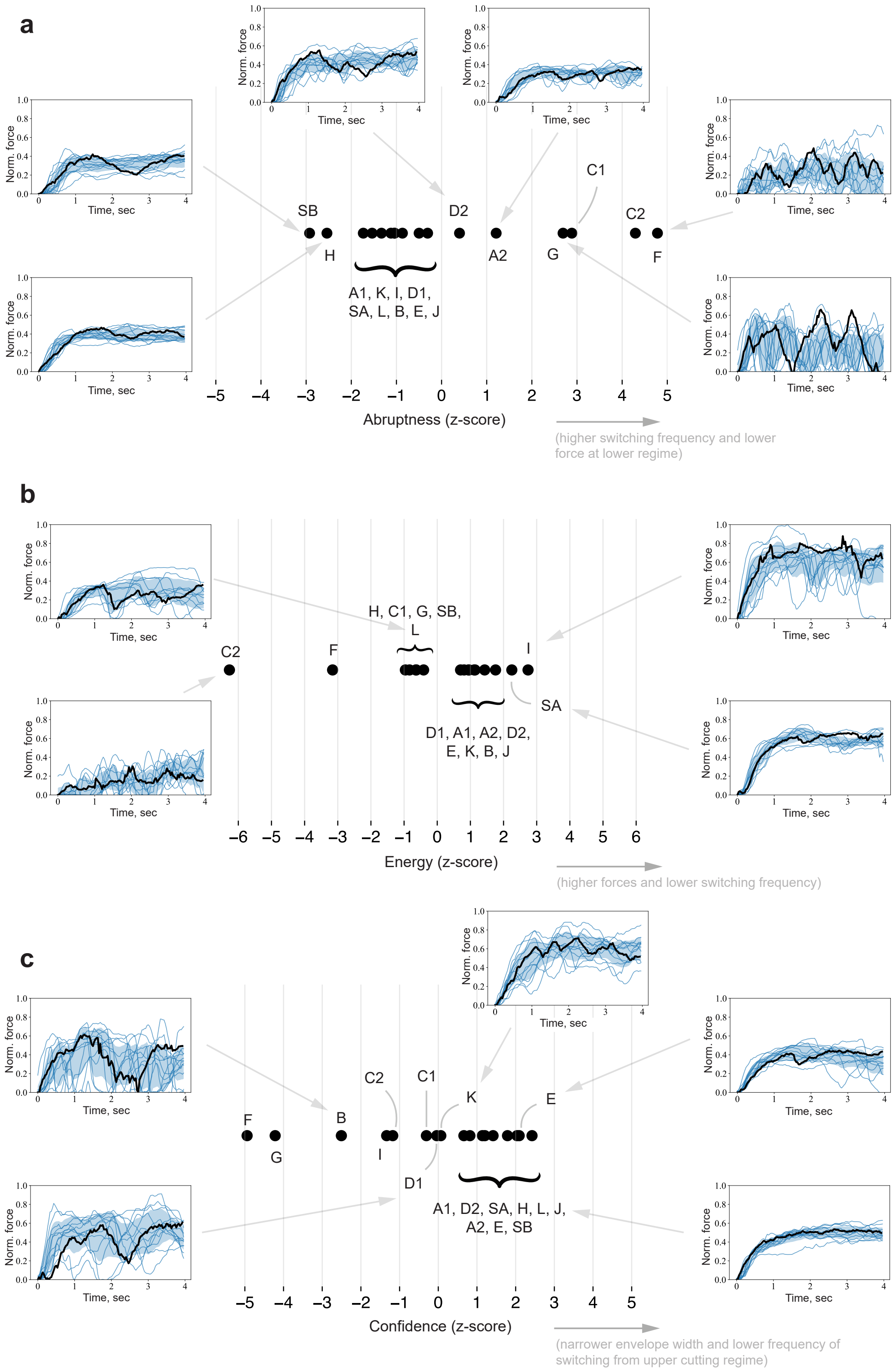

Supplementary Figure 8: **Projection of latent parameter space on feature axes**. **a** Abruptness feature, **b** Energy feature, and **c** Confidence feature. Blue lines are the actual force measurements, blue regions are the standard deviation of recorded force profiles ($N = 12$ independent trials), and black lines are (generated) force profiles sampled from the learned model. Note: A-L letters indicate the force profiles for medical students, SA and SB labels correspond to the force profiles for surgeon A and surgeon B, respectively.

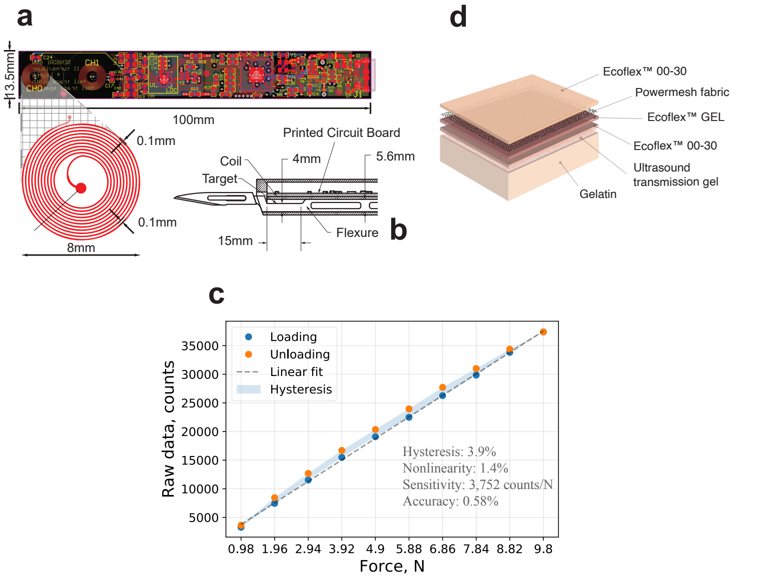

Supplementary Figure 9: **Sensorised instrument and skin tissue phantom.** **a** PCB layout design with planar 8.6 μH coils (8 mm diameter, 11 turns per each of four layers, 0.1 mm trace width and 0.1 mm spacing). **b** Cross-sectional schematic of the cutting tool and flexure dimensions. **c** Incremental loading test results. **d** Design and material composition of the skin-mimicking phantom.
