# Supplementary material for: A generative force model for surgical skill quantification using sensorised instruments
Source: Commun Eng. 2023 Jun 10;2:36. doi: 10.1038/s44172-023-00086-z (PMC10955904; doi:10.1038/s44172-023-00086-z)
Supplement: Supplementary file 3 — Description of Additional Supplementary Files [file 44172_2023_86_MOESM3_ESM.pdf]

Description of Additional Supplementary Files

File name: Supplementary Movie

Description: The experiment. 12 medical students performing the elliptical excision task. Note: Subjects A, C and D have repeated the trials.
